# Visual Target Strategies in Infantile Nystagmus Patients With Horizontal Jerk Waveform

**DOI:** 10.3389/fneur.2018.00622

**Published:** 2018-07-30

**Authors:** Takao Imai, Yasumitsu Takimoto, Tomoko Okumura, Kayoko Higashi-Shingai, Noriaki Takeda, Koji Kitamura, Bukasa Kalubi, Takashi Fujikado, Masakazu Hirota, Yoshihiro Midoh, Koji Nakamae, Hidenori Inohara

**Affiliations:** ^1^Department of Otorhinolaryngology - Head and Neck Surgery, Osaka University Graduate School of Medicine, Osaka, Japan; ^2^Department of Otolaryngology, Osaka Police Hospital, Osaka, Japan; ^3^Department of Otolaryngology, Yao Municipal Hospital, Osaka, Japan; ^4^Department of Otorhinolaryngology - Head and Neck Surgery, Tokushima University Graduate School of Medicine, Tokushima, Japan; ^5^Medical Education Center, Osaka University Graduate School of Medicine, Osaka, Japan; ^6^Department of Ophthalmology, Osaka University Graduate School of Medicine, Osaka, Japan; ^7^Department of Information Systems Engineering, Osaka University Graduate School of Information Science and Technology, Osaka, Japan

**Keywords:** infantile nystagmus, saccade, three-dimensional analysis, optokinetic nystagmus, vestibulo-ocular reflex

## Abstract

The aim of this study was to propose a new pathophysiological hypothesis for involuntary eye oscillation in infantile nystagmus (IN): patients with IN exhibit impaired gaze fixation, horizontal smooth pursuit and optokinetic nystagmus (OKN) and use saccadic eye movements for these underlying impairments. In order to induce saccades, they make enough angle between gaze and target by precedent exponential slow eye movements. IN consists of the alternate appearance of the saccade and the slow eye movements. Unlike most previous theories, IN is therefore considered a necessary strategy allowing for better vision and not an obstacle to clear vision. In five patients with IN, eye movements were analyzed during the smooth pursuit test, saccadic eye movement test, OKN test and vestibulo-ocular reflex (VOR) test. Their gaze fixation, horizontal smooth pursuit, OKN and the last half of the slow phase of VOR were impaired. The lines obtained by connection of the end eye positions of fast phase of nystagmus coincided with the trajectories of targets. The findings indicate that patients followed the target by the fast but not the slow phase of nystagmus, which supports our hypothesis. By setting the direction of slow phase of nystagmus opposite to the direction of the OKN stimulation, enough angle can be effectively made between the gaze and target for the induction of saccade. This is the mechanism of reversed OKN response. In darkness and when eyes are closed, IN weakens because there is no visual target and neither the saccade for catching up the target or slow phase for induction of the saccade is needed.

## Introduction

Patients with congenital ocular oscillations show three distinct syndromes: infantile nystagmus (IN), fusion maldevelopment nystagmus syndrome, and spasmus nutans syndrome (1). IN is also characterized by waveforms, the most common of which is the horizontal jerk type where horizontal slow phases exhibit increasing velocity exponentials (2). This type of nystagmus exhibits two unusual characteristics that appear to impair visual function. First, IN is usually accentuated when one attempts to fixate upon an object (3), but eyelid closure usually suppresses the nystagmus (4). Second, patients may exhibit reversed optokinetic nystagmus (OKN) response, where the slow phase direction opposes that of the stimulus movement (5). Although many oculo-motor subsystems have been suggested to be the origin of these two unusual characteristics (6, 7), the exact origin is still controversial. Previous theories were based on the concept that IN is an unnecessary eye oscillation that obstructs clear vision. In this study, we reverse this theory and show that IN is actually a necessary visual strategy for these patients because of their disturbed gaze fixation and horizontal smooth pursuit functions. Our novel theory holds that patients use saccadic eye movements to compensate for the disturbances. In order to induce saccades, they make enough angle between gaze and target by precedent exponential slow eye movements. IN consists of the alternate appearance of the saccade and the slow eye movements. Our novel theory explains how IN patients with horizontal jerk waveform view visual targets, and why (1) attempts to fixate upon an object accentuate nystagmus that is suppressed in darkness. (2) These patients exhibit reversed OKN response. More, we demonstrate how their visuo-vestibular system performs during rotation in light.

## Materials and methods

From December 2012 to May 2017, five IN patients with horizontal jerk waveform (four women and a man; 13–52 years of age, median 33 years) visited our department complaining of abnormal eye movement. No patients had ocular albinism. Ophthalmologic and neurologic examinations were unremarkable except for gaze nystagmus and reversed OKN response. The study inclusion criteria were: (1) Presence of a horizontal jerky type gaze nystagmus. (2) No complaint of oscillopsia. (3) No head oscillation. (4) Good visual acuity when viewing with their null zone (i.e., weak nystagmus while looking at an object (8) during a visual acuity test. (5) Presence of reversed OKN response. All patients were assessed for smooth pursuit, saccadic eye movements, optokinetic nystagmus, visual fixation, vestibulo-ocular reflex (VOR) in darkness and visuo-vestibular interaction in light during rotation. In addition, five healthy participants (3 women and 2 men; 29–60 years, median 31 years) were recruited as control.

This study was approved by the ethics committee of Osaka University Hospital (Nos. 10091, 12095), registered under the University Hospital Medical Information Network (study IDs: UMIN000010683, UMIN000020047), and performed in accordance with the Declaration of Helsinki. Before examination, a written informed consent was obtained from all patients and healthy participants.

### Saccadic eye movement, smooth pursuit, optokinetic nystagmus, and visual fixation test

Visual targets were projected onto a white cylindrical screen of 160-cm radius (comprehensive balance function examination device, MVM-C2, NAGASHIMA MEDICAL INSTRUMENTS CO., Tokyo, Japan) using a projector (ELP-73, EPSON, Tokyo, Japan). Visual stimuli were created using Visual Studio 2010 (MICROSOFT, Washington, D.C., USA) on a Windows-operated computer. Participants sat on a chair located 100 cm away from the screen. The back of each participant's head was firmly secured to a headrest via pads and adjustable clamps cushioned with stiff conforming foam (BIO-MEDICA, Osaka, Japan).

During the saccadic eye movement test, a target was presented to either the left or right of the patient's gaze. The target was a red circle of 8-mm diameter. Initially, the target appeared and jumped to the right and left randomly. During the smooth pursuit test, the same target was moved sinusoidally at 0.1, 0.2, and 0.3 Hz, with a maximum angular velocity of 5, 10, and 20°/s horizontally, vertically, and obliquely. During the optokinetic nystagmus test, optokinetic stimuli were presented on the screen subtending 82° horizontally and 55° vertically. The stimulus consisted of a projected random dot pattern, with each dot subtending a diameter between 1.5 and 6.5°, thus resulting in a dot density of 70 dots/m^2^. Two stimulus velocities were used, 5 and 8°/s, in rightward, leftward, upward, downward, and diagonal directions. The OKN stimulation was also moved horizontally and sinusoidally at frequencies of 0.2 and 0.3 Hz and at a maximum angular velocities of 10 and 20°/s. For the visual fixation test, the red circle target randomly appeared on the screen, then disappeared in light. A static random dots pattern was also presented, which then disappeared in both light and dark conditions. During all tests, participants were asked to fixate upon moving or still visual targets, with each trial duration lasting 40 s.

### Vestibulo-ocular reflex and visuo-vestibular interaction test

During the VOR test in darkness, participants sat upright in a chair with their head secured to the headrest. In complete darkness, participants were rotated on a computer-controlled chair (DAIICHI MEDICAL CO., Tokyo, Japan) (9). Before rotation, participants were presented with the same visual target that was used for all tests (the red circle of 8-mm diameter). They were instructed to imagine the target and to perform a mental arithmetic calculation during rotation. During the visuo-vestibular interaction test in light, participants sat upright on the chair, with their head secured to the headrest. They were rotated while seated on the chair in light within the cylindrical white screen. During rotation, a static black-and-white-striped pattern generated by the MVM-C2 was projected on the screen. During rotation, participants were asked to fixate upon the stripes.

During both the VOR test and the visuo-vestibular interaction test, the chair was rotated in line with the Earth's vertical axis sinusoidally at 0.1 Hz with a maximum angular velocity of 40°/s, and at 0.3 Hz with a maximum angular velocity of 50°/s. The duration of each trial was 40 s. To analyze chair movement during rotation, two markers on the ceiling were recorded at 30 Hz with a camcorder (DCR-PC101K, SONY, Tokyo, Japan) secured to the chair (9).

### Recording and analysis of the three-dimensional eye movement

During all tests other than rotation in darkness, the participants wore goggles (BIO-MEDICA), to which a 240-Hz digital camera (STC-CL338A, SENTECH CO., Kanagawa, Japan) was attached. Also attached to the goggles was a semi-transparent mirror that was positioned in front of the patient's left eye without obstructing their view. The camera recorded the left eye's images that were reflected by the semi-transparent mirror. During rotation in darkness, they wore goggles equipped with the digital camera, with an additional infrared luminous source positioned in front of their left eye (BIO-MEDICA, Osaka, Japan). Left eye movements were recorded and saved to a computer hard disk using software (StreamPix; NorPix Inc, Montreal, Canada). During the eye movement recording the image displayed on the computer screen was converted into analog output signal that was transmitted to a color quad processor (SG-202II, DAIWA, Tokyo, Japan). Except for the VOR and visuo-vestibular interaction tests, all the visual stimuli were displayed on the computer screen and their images converted into analog output and the analog signals transmitted to the color quad processor. During the VOR and visuo-vestibular interaction tests, the chair's movement recorded by a camcorder was transmitted to the color quad processor. By using the image of the color quad processor, we synchronized eye movement data with those of the visual stimulation or the chair movement (9). Additionally, 3-dimensional (3D) eye movements were described using rotation vectors, i.e., 3D eye positions of a single rotation (10). Analysis of eye rotation vectors using this method and the accuracy of this method have been described previously (11–14). Two hundred and forty Hz 640- × 485-dot JPG images were obtained using StreamPix, and from them, we analyzed eye rotation vectors. The X, Y, and Z components reflect the roll, pitch, and yaw components, respectively. We used Euler angle at 2 × tan^−1^ (rotation vector magnitude) to determine the eye position as axis–angle representations (15). We extracted data on the slow-phase eye velocity (SPEV) from data on the nystagmic eye movement by applying a method based on a fuzzy-set approach (16, 17).

## Results

**Figure 1:**
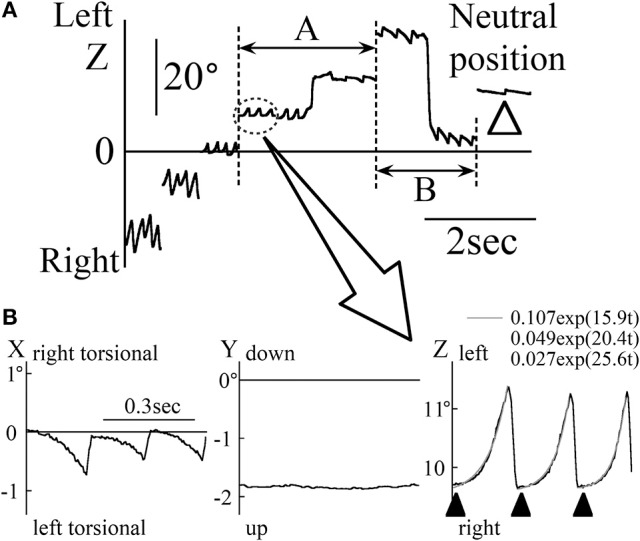
The eye movement data of patient 1 during the horizontal saccadic eye movement test. **(A)** The Z component of the eye position data. White arrow heads show when the eye was at an approximately 20° leftward position. At this point, the nystagmus was very weak and the eye was in null zone. When eye was to the right of the null zone, rightward nystagmus was observed, and when the eye was to the left of the null zone, leftward nystagmus was observed. **(B)** Three-dimensional eye position data of the part enclosed by dotted ellipse in **(A)**. The slow phase of nystagmus in the Z component was exponential. The gray lines in the Z component were also exponential, as obtained by exponential approximation to the slow phase of nystagmus. Black arrow heads in the Z component show the foveation period. At the foveation period, X component was also relatively stationary.

Results of the eye movement of patient 1 (33-year-old man) during the saccadic eye movement test are shown in Figure 1. When looking at a target approximately 20° rightward, the patient's eye position was in the null zone and his gaze intensity evoked only a very small nystagmus (white arrow head in Figure 1A). The direction of the slow phase of eye movement was toward the null zone. Therefore, when the eye position changed across the null zone, the nystagmus direction also changed (see part A of Figure 1A). However, it rarely happens that the nystagmus is unchanged when the eye position changes across the null zone as shown in part B of Figure 1A in patient 1. When the eye position was away from the null zone, the nystagmus was intense (see Figure 1A). Figure 1B shows the three-dimensional eye position data obtained by magnifying the part enclosed by dotted ellipse of Figure 1A. Torsio-horizontal nystagmus (X and Z components) was observed, with no vertical component (Y component). This nystagmus was either rightward and right torsional or leftward and left torsional. As in past studies (18, 19), the Z component of the slow phase was exponential (gray lines) and the waveform containing the foveation period, the eyes were relatively stationary (black arrow heads in the Z component of Figure 1B). During the foveation period, the X component (torsional component) was also stationary. Although the null zone differed among patients, the other four patients displayed the same characteristic as described above.

**Figure 2:**
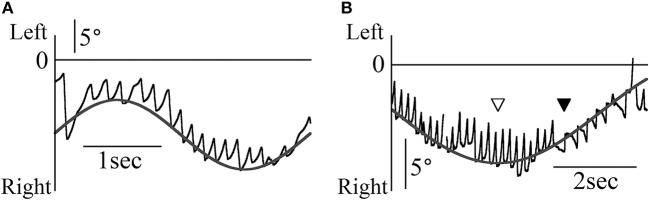
The Z component of patient 1's eye data during the horizontal smooth pursuit test. **(A)** The Z component of the eye position data when the target moved sinusoidally and horizontally at 0.3 Hz. The direction of nystagmus remained constant despite changes in the target's direction. The end point of the fast phase of each nystagmus followed the gray line representing the target's movement. This eye movement can be observed in Movie 1 attached to this paper. **(B)** The Z component of the eye position data when the target moved sinusoidally and horizontally at 0.1 Hz. Although the target direction changed at the point shown by the white arrow head, the direction of nystagmus changed at the point shown by the black arrow head. Regardless of the direction of nystagmus, the end point of the fast phase of each nystagmus followed the gray line representing the target's movement.

Figure 2A shows patient 1's eye movement in the Z component (black line) during the smooth pursuit test when the target moved sinusoidally and horizontally at 0.3 Hz and maximum angular velocity of 10°/s. The direction of nystagmus remained constant despite changes in the target's direction. The target position was determined by the formula 10/(2π·0.3)sin(2π·0.3 t)+α (where t is time). By determining α, we could draw the gray line of the formula on all end points of the fast phase of each nystagmus (Figure 2A). However, the direction of nystagmus sometimes changed during one trial of the horizontal smooth pursuit test (Figure 2B). The data were obtained when patient 1 completed the smooth pursuit test in which the target moved sinusoidally and horizontally at 0.1 Hz and at a maximum angular velocity of 10°/s. The target position was determined by the formula 10/(2π·0.1)sin(2π·0.1 t)+β. By determining β, we could draw the gray line of the formula on all end points of the fast phase of each nystagmus (Figure 2B). The point where the direction of target movement changed (white arrow head) was different from that where the direction of nystagmus changed (black arrow head). The phase difference between these two points was approximately 90° of one full cycle (360°). Thus, the direction of nystagmus reversed when the target that moved leftward reached maximum velocity. In the other four patients, we could draw the line of the sine formula of which frequency and amplitude were same as the sine formula of stimulation on all end points of the fast phase of each nystagmus.

**Figure 3:**
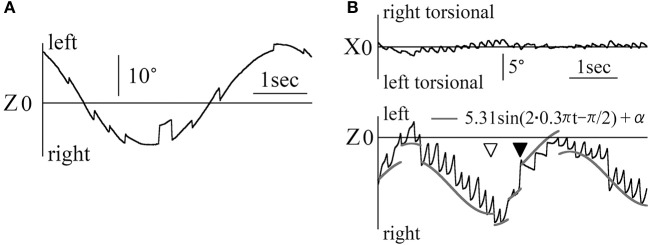
The eye position data during the OKN test for which random dots moved sinusoidally and horizontally **(A)** Healthy participant 1's eye position data during the OKN test for which random dots moved at 0.2 Hz. Eye movement formed the shape of nystagmus. **(B)** Patient 1's eye position data during the OKN test for which random dots moved at 0.3 Hz. Rightward and right torsional nystagmus, and leftward and left torsional nystagmus was observed. By determining γ of 10/(2π·0.3)sin(2π·0.3 t)+ γ, we could draw the gray line of the formula on end points of the fast phase of each nystagmus. The healthy participant followed the movement of one pattern of random dots via one slow phase of nystagmus. However, the patient followed the movement of the same pattern by multiple fast phases of nystagmus.

In Figure 3A, the Z component of a 60-year old healthy woman's eye position data are shown for the OKN test, where random dots moved sinusoidally and horizontally at 0.2 Hz at a maximum angular velocity of 20°/s, and nystagmus was observed. Also, nystagmus was observed in the other four healthy participants. In Figure 3B, the X and Z components of the eye position data are shown for patient 1, who completed the OKN test, for which random dots moved sinusoidally and horizontally at 0.3 Hz at a maximum angular velocity of 10°/s. Here also, either rightward and right torsional nystagmus or leftward and left torsional nystagmus was observed. The position of the random dots were determined by 10/(2π·0.3)sin(2π·0.3 t)+γ. We attempted to draw curved lines of the formula by setting the value of γ so that the end points of the fast phase of nystagmus lay on the curved lines (gray curved lines in Figure 3B). The gray curved lines closely followed the Z component of healthy participants' eye position, as shown in Figure 3A. When gazing at sinusoidal and horizontal OKN stimuli, the horizontal direction of the slow phase of nystagmus was not always opposed to that of OKN stimulation despite exhibiting a reversed OKN response (showing reversed OKN response is one of this study inclusion criteria and the data are shown below). As in the eye movement during the horizontal and sinusoidal smooth pursuit test (Figure 2B), the phase difference between the point at which the horizontal direction of nystagmus (black arrow head) and that of the random dots (white arrow head) changed was approximately 90°. In the other four patients, we could draw curved lines of the sine formula of which frequency and amplitude were the same as the sine formula of the stimulation on all end points of the fast phase of each nystagmus and the direction of the slow phase was not always opposite to the direction of movement of OKN stimulation.

**Figure 4:**
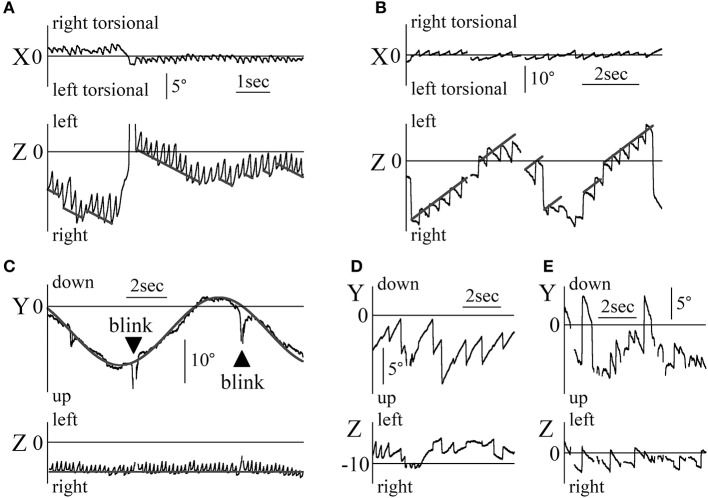
Patients' eye movement data with reversed OKN response and eye movement data during the vertical smooth pursuit test and the vertical OKN test **(A)** Eye movement data when patient 1 gazed at random dots that moved rightward horizontally at a constant velocity of 5°/s. Right torsional and rightward nystagmus was observed. The response was reversed OKN response. The end points of the fast phase of nystagmus were connected, and the resulting incline (gray) was the same angular velocity as the angular velocity of the OKN stimulation. **(B)** Eye movement data when patient 2 gazed at random dots that moved leftward horizontally at a constant velocity of 8°/s. Left torsional and leftward nystagmus was observed. The response was reversed OKN response. The end points of the fast phase of nystagmus were connected, resulting in an incline (gray) that was the same angular velocity as that for the angular velocity of the OKN stimulation. **(C)** Patient 1's eye position data during the vertical smooth pursuit test for which the target moved sinusoidally vertical at 0.1 Hz. By determining δ of 5/(2π·0.1)sin(2π·0.1 t)+δ, gray line was drawn on the data of the Y component of the eye. In the Z component, rightward nystagmus existed despite the coordinate of the lateral direction of the target being constant. The value of the coordinates of the Z component at the end points of the fast phase for each nystagmus was almost constant (gray line in the Z component). This eye movement can be observed in Movie 2 attached to this paper. **(D)** Patient 1's eye position data during the vertical OKN test for which random dots moved downwards at a constant velocity of 5°/s. In the Y component, upward nystagmus was observed. That is, during the vertical OKN test, reversed OKN response was not observed. Although the random dots did not move laterally, in the Z component, nystagmus was observed, and the direction of most of the nystagmus was rightward because the eye position was on the right side, against the patient's null zone. **(E)** Patient 2's eye position data during the vertical OKN test for which random dots moved upwards at a constant velocity of 8°/s. In the Y component, downward nystagmus was observed. That is, during the vertical OKN test, the similarity with data shown in **(D)** was evident, where the reversed OKN response was not observed. Although the random dots did not move laterally, in the Z component, nystagmus was observed and the direction of nystagmus was always leftward because the eye position was to the left of her null zone.

Figure 4A and B shows the X and Z components of eye position data during the OKN test where random dots moved horizontally at a constant angular velocity (Figure 4A: patient 1, 5°/s rightward OKN stimulation, Figure 4B: patient 2 (37-year-old woman), 8°/s leftward OKN stimulation). Reversed OKN response, one of our inclusion criteria, was observed in these patients. The direction of nystagmus was either rightward and right torsional or leftward and left torsional. When the end points of the fast phase of each nystagmus were connected, the line (gray) of the resulting slope was almost the same as that of the angular velocity of OKN stimulation (the inclination was 5.08°/s in Figure 4A, and 8.13°/s in Figure 4B). In the other three patients, when the end points of the fast phase of each nystagmus were connected, the line of the resulting slope was almost the same as that of the angular velocity of OKN stimulation.

Figure 4C shows the Y and Z components of the eye movement of patient 1 during the smooth pursuit test where the target moved purely sinusoidally and vertically at 0.1 Hz, at a maximum angular velocity of 5°/s. The target position of the Y (vertical) component was determined by 5/(2π·0.1)sin(2π·0.1 t)+δ. For this Y component, although the movement was slightly rough, by determining δ, the eye movement could be superimposed on the target movement (gray line). For the Z component (horizontal component), although the target did not move in a horizontal direction at all, the eye movement formed nystagmus, which was always rightward because the eye position was always to the right of his null zone. The position of the Z component at the end point of the fast phase of the nystagmus was almost constant (gray line). In the other four patients, the Y component of eye movement followed the vertical movement of the target although the Y component was slightly rough. In Z component, the nystagmus could be seen and the position of the Z component at the end point of the fast phase of the nystagmus was almost constant.

Figures 4D,E shows the Y and Z components of the eye position data during the OKN test where random dots moved in a purely vertical direction at a constant angular velocity Figure 4D: patient 1, 5°/s downward OKN stimulation, Figure 4E: patient 2, 8°/s upward OKN stimulation). For the Y component, the direction of the slow phase of nystagmus was the same as that of healthy participants, i.e., normal, and reversed OKN responses were not observed. For the Z component, nystagmus was observed, and results differed from those of healthy participants. In the other three patients, in Y component, normal OKN response, not reversed OKN response could be seen, and in Z component, nystagmus was observed.

**Figure 5:**
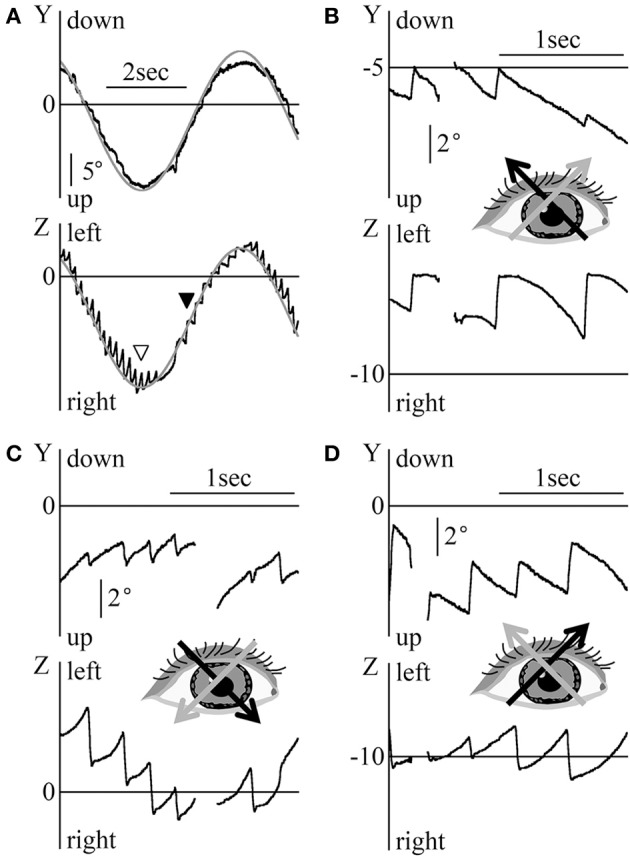
Eye position data during the diagonal smooth pursuit and OKN tests **(A)** Patient 1's eye position data during the diagonal smooth pursuit test for which the target moved sinusoidally between the upper right corner and the lower left corner of the screen at 0.2 Hz and at a maximum velocity of 20 °/s. By determining ε of $20/\sqrt{2}/\left(2\pi \cdot 0.2\right)\text{sin}\left(2\pi \cdot 0.2\text{t}\right)+\varepsilon$, the gray line was drawn on the data of the Y component of the eye. By determining ζ of $20/\sqrt{2}/\left(2\pi \cdot 0.2\right)\text{sin}\left(2\pi \cdot 0.2\text{t}\right)+\zeta$, the end of the fast phases followed the gray line. The point shown by the white arrow head was that at which the target's direction changed from rightward to leftward. The point shown by the black arrow head was that at which the horizontal direction of nystagmus changed from right to left. **(B–D)** Patient 2's eye position data during the diagonal OKN test at a constant velocity of 8°/s; **(B)** The random dots moved from the lower right to the upper left of the screen. **(C)** The random dots moved from the upper left to the lower right of the screen. **(D)** The random dots moved from the lower left to the upper right of the screen. The direction of the black arrow for the inserted eye figures shows the direction of eye movement during the slow phase of nystagmus. The direction of the gray arrow shows the direction of random dots movement. The Y component of the normal OKN response and the Z component of the reversed OKN response could be seen.

Figure 5A shows the eye movement of patient 1 during the smooth pursuit test for which the target moved obliquely, thus forming a slanted angle of 45° between the upper right and the lower left of the screen sinusoidally at 0.2 Hz, at maximum angular velocity of 20°/s. For the Y component, the eye moved almost simultaneously with the target's movement, i.e., the eye position data could be superimposed on the target's vertical movement, as shown by the gray line, by the formula $20/\sqrt{2}/\left(2\pi \cdot 0.2\right)\sin\left(2\pi \cdot 0.2t\right)+\varepsilon$, where ε was determined. For the Z component, nystagmus was observed and the end point of the fast phase of nystagmus followed the target's horizontal movement, as shown by the gray line, by the formula $20/\sqrt{2}/\left(2\pi \cdot 0.2\right)\sin\left(2\pi \cdot 0.2t\right)+\zeta$, where ζ was determined. Similarly to Figures 2B, 3B, the difference between the point of the target's horizontal movement and that of the horizontal direction of nystagmus changed by 90°. In the other four patients, the Y component of eye movement followed the vertical movement of the target although the Y component was slightly rough. In Z component, we could draw the line of the sine formula of which frequency and amplitude were the same as the sine formula of Z component of stimulation on all end points of the fast phase of each nystagmus.

Figures 5B–D shows patient 2's eye position data during the OKN test where random dots moved obliquely at a constant angular velocity of 8°/s. The Y component direction of nystagmus in patient 2 was identical to that of nystagmus in healthy participants. However, the direction of the Z component was opposed to that of nystagmus in healthy participants. That is, a reversed OKN response could only be observed in the Z component (figure inserts; gray arrow shows the direction of movement of random dots and black arrow shows the direction of slow phase eye movement). The results of the oblique OKN test for the other four patients were similar to those of patient 2, the Y component of the normal OKN response and the Z component of the reversed OKN response could be observed.

**Figure 6:**
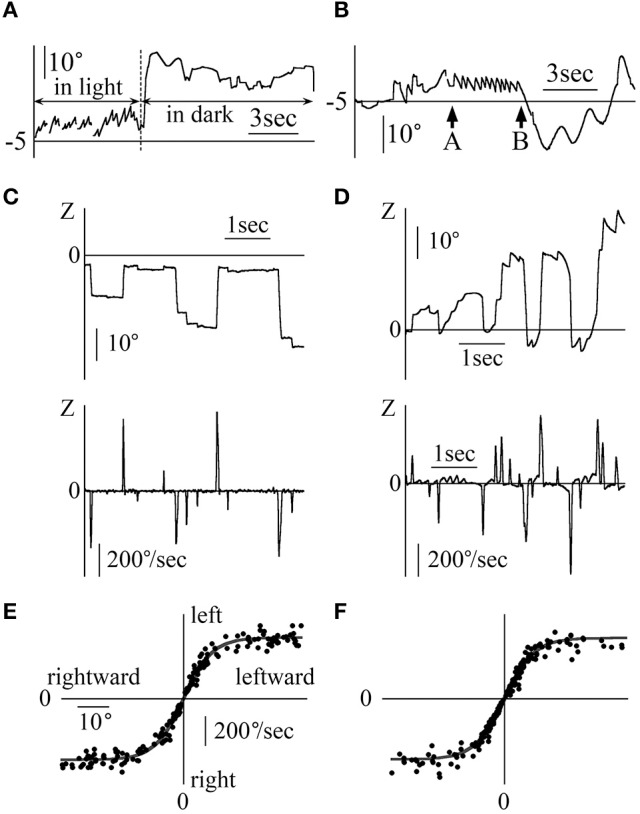
Eye movement data in light and in darkness **(A)** The Z component of the eye movement data when patient 3 gazed at a still random dots pattern in light and in darkness during a fixation test. In light, rightward nystagmus was observed, but when the room went black, no clear nystagmus could be observed. **(B)** The Z component of the eye movement data when patient 3 looked at a white screen in light during the fixation test. In light, during part A, a small red circle target was presented on the screen; during part B, the target was removed. During transition between A and B, leftward nystagmus was observed, but during the other parts, no clear nystagmus was observed, even in light condition. **(C)** Z component of healthy participant 2's eye movement data during the horizontal saccadic eye movement test. The upper graph displays the eye position data. The lower graph shows the eye velocity data. No nystagmus was observed. When the amplitude of the saccadic eye movement was large, the eye velocity was also large. **(D)** Z component of patient 1's eye movement data during the horizontal saccadic eye movement test. The upper graph displays the eye position data, and the lower graph shows the eye velocity data. Nystagmus was observed. **(E)** Relationship between the amplitude of the horizontal saccadic eye movement and the horizontal eye velocity during the horizontal saccadic eye movement test in five healthy participants. The abscissa of the graph represents the amplitude of the horizontal saccadic eye movement, while the ordinate represents the horizontal eye velocity. The relationship formed a sigmoid curve. **(F)** The relationship between the amplitude of the horizontal saccadic eye movement and the horizontal eye velocity during the horizontal saccadic eye movement test in five patients. The relationship formed almost the same sigmoid curve as that shown in **(E)**.

Figures 6A,B shows the eye movements in the Z component of patient 3 (a 25-year-old woman) during the fixation test. Figure 6A displays her eye position data when gazing at stationary random dots that disappeared when the room suddenly went black. In the Z component, leftward nystagmus was observed in light but not in darkness. Figure 6B shows the eye position data with the patient in light conditions, facing the white screen that initially had no target, and then suddenly showed a single red circle target, which disappeared again. In absence of target, no nystagmus was seen. When a single red circle target appeared on the screen, regular nystagmus appeared. Finally, when the target disappeared from the screen, no nystagmus was observed despite the light conditions. In the other four patients, when there was a target, regular nystagmus could be seen, but when there was no target or in darkness, a weak irregular or no nystagmus could be seen.

Figure 6C shows the eye angular Z component position and velocity data of a healthy 44-year-old man during the horizontal saccadic eye movement test. The maximum velocity of saccadic eye movement varied (lower column in Figure 6C) proportionally to the amplitude of saccadic eye movement (upper vs. lower column in Figure 6C). Figure 6D displays the Z component of the eye's angular position and the velocity data of patient 1 during the same saccadic eye movement test. As in healthy participants, the velocity of saccadic eye movement was proportional to its amplitude. Using the horizontal saccadic eye movement test data of all five healthy participants (Figure 6E) and five patients (Figure 6F), amplitudes of saccadic eye movement were plotted on an abscissa axis and values of the maximum angular velocity on the vertical axis. Figure 6F was similar to Figure 6E.

**Figure 7:**
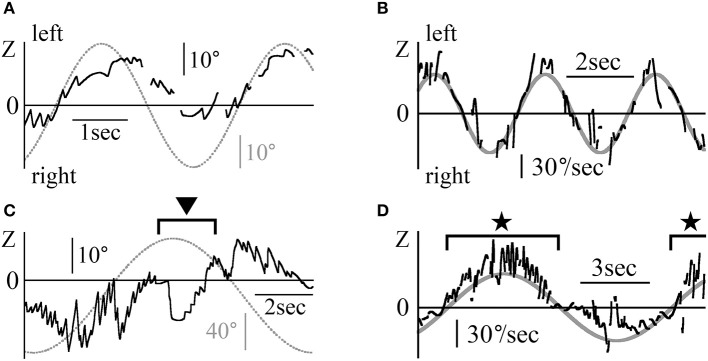
The Z component of patients' eye movement data during the VOR test when rotating in darkness **(A)** The Z component of patient 2's eye position data during rotation in darkness.The patient was rotated sinusoidally at 0.3 Hz and at a maximum velocity of 50°/s in darkness. The dotted gray line represents the relative movement of the scene (50/(2π·0.3)sin(2π·0.3t)). **(B)** Slow phase eye velocity data obtained by differentiation of the data shown in **(A)**. The gray line represents the relative velocity of the scene movement. The initial velocity of the slow phase of each nystagmus almost followed the gray line. The chair-velocity relative to space was 180° out of phase with the gray line. **(C)** Z component of patient 1's eye position data during rotation in darkness. The patient was rotated sinusoidally at 0.1 Hz and at a maximum velocity of 40°/s in darkness. Dotted gray line represents the relative movement of the scene (40/(2π·0.1)sin(2π·0.1t)). Nystagmus was weaker during leftward rotation at the part shown by black arrow heads as compared to rightward rotation. **(D)** Slow phase eye velocity data obtained by differentiation of the data shown in **(C)**. The gray line represents the relative velocity of the scene movement. The initial velocity of the slow phase of each nystagmus closely followed the gray line when rotating rightward (black stars). However, when rotating leftward, the slow phase eye velocity was very low.

Figure 7 shows the Z component of the eye data during the VOR test in darkness. The rotational frequencies and maximum angular velocities were respectively 0.3 Hz and 50°/s for patient 2 (Figures 7A,B), and 0.1 Hz and 40°/s for patient 1 (Figures 7C,D). Figures 7A,C shows the position data, while Figures 7B,D show the slow phase angular velocity data of the eye and the relative movement of the outside world (dotted and solid gray lines). Figures 7A,C show how the eye movement attempted to catch up with the relative outside world movement except for the part shown by the black arrow head in Figure 7C. That is, their VOR in darkness was functioning normally. The starting point of slow phase of each nystagmus almost followed the sine curve of the angular velocity of the relative outside world movement (gray line, 50sin(2π·0.3t) in Figure 7B and 40sin(2π·0.1t) in Figure 7D), and then the slow phase eye velocity changed dramatically for the whole of Figure 7B and the portion shown by black stars in Figure 7D. The eye movement was weak in the portion shown by the black arrow head in Figure 7C and in other portions of Figure 7D except those indicated by black stars. In the other three patients, the starting point of slow phase of each nystagmus almost followed the sine curve of the angular velocity of the relative outside world movement.

**Figure 8:**
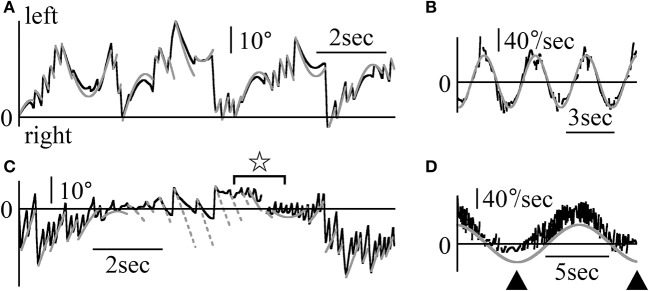
Z component of patients' eye movement data during the visuo-vestibular interaction test when rotating in light **(A)** Z component of patient 2's eye position data when rotating in light. The patient was rotated sinusoidally at 0.3 Hz and at a maximum velocity of 50°/sin light. By determining η from 50/(2π·0.3)sin(2π·0.3t)+η that represents the relative movement of the scene, gray lines were drawn to agree with the formula of the Z component of the slow phase of each nystagmus at its starting point. Eye movement almost followed the gray lines. **(B)** Slow phase eye velocity data obtained by differentiation of the data shown in **(A)**. The gray line represents the relative velocity of the scene movement. The initial velocity of the slow phase of each nystagmus closely followed the gray line. **(C)** The Z component of patient 1's eye position data during rotation in light. The patient was rotated sinusoidally at 0.1 Hz and at a maximum velocity of 40°/s in darkness. By determining θ of 40/(2π·0.1)sin(2π·0.1t)+θ that represents the relative movement of the scene, solid and dotted gray lines were drawn to agree with the formula of the Z component of the slow phase for each nystagmus at the start point of the slow phase of each nystagmus. When rotating rightward, as shown by solid gray lines, several end points of the fast phase of nystagmus could be represented by the formula. However, for leftward nystagmus, as shown by dotted lines, only one end point of the fast phase was determined by the formula. At the part shown by star, the direction of slow phase eye movement was opposite to the direction of the relative movement of the scene. **(D)** Slow phase eye velocity data obtained by differentiation of the data are shown in **(C)**. The gray line represents the relative velocity of the scene movement. The initial velocity of the slow phase of each nystagmus closely followed the gray line when rotating rightward. However, during leftward rotation, the slow phase eye velocity was very low (shown by black arrow heads).

Figure 8 shows the Z component of the eye data during the visuo-vestibular interaction test in light conditions. The rotational frequencies and maximum angular velocities were respectively 0.3 Hz and 50°/s for patient 2 (Figures 8A,B), and 0.1 Hz and 40°/s for patient 1 (Figures 8C,D). In Figure 8A, the position of the relative movement of the outside world was determined by the formula, 50/(2π·0.3)sin(2π·0.3 t)+η. By determining η, the patient's eye data followed the formula (gray lines, Figure 8A) regardless of the direction of rotation. When calculating the slow phase eye velocity (Figure 8B), the starting point of each nystagmus almost followed the sine curve of the angular velocity of the relative outside world movement (gray line). Additionally, the slow phase eye velocity changed dramatically, similarly to the eye velocity during VOR in darkness as shown in Figure 7B. In Figure 8C, the position of the moving scene was determined by the formula, 40/(2π·0.1)sin(2π·0.1 t)+θ. When rotating rightward, and upon determining θ, several end points of the fast phase of nystagmus could be represented by the formula (solid gray line, Figure 8C). These results indicate that the patient could catch the relative movement of the outside world by using the fast phase of nystagmus, i.e., the saccadic eye movement. Furthermore, after catching up with the movement of the outside world, the patient pursued the movement by following the first part of the slow phase of nystagmus. Then, his gaze moved away from the movement of the outside world, and he could catch again the scene's movement by using the fast phase of nystagmus. However, for leftward nystagmus, only one end point of the fast phase was determined by the formula (dotted gray line, Figure 8C). This result suggests that during leftward nystagmus, the patient caught the relative movement of the outside world by using the fast phase of nystagmus. That is, he used saccadic eye movement, and after catching up with the scene, he was unable to follow the movement by using the slow phase of nystagmus. In practice, he stated that he could follow the scene's movement when rotating rightward better than when rotating leftward. When calculating the slow phase eye velocity (Figure 8D), when rotating rightward, the starting point of each nystagmus almost followed the sine curve of the angular velocity of the relative outside world movement. But, when rotating leftward, the value of the angular velocity was kept almost constanly low as shown by black arrowheads of Figure 8D. All patients stated that they could follow the scene movement when rotating to one side better than to the other (e.g., patient 2 could follow scene movement better when rotating leftward). In the other three patients, the starting point of slow phase of each nystagmus followed the sine curve of the angular velocity of the relative outside world movement and the slow phase eye velocity changed dramatically.

## Discussion

We proposed a new theory on the cause of IN with a horizontal jerk waveform. In these patients' gaze fixation (Figure 1), horizontal smooth pursuit (Figure 2) and horizontal OKN (Figure 3B, Figure 4A,B) are disordered. To compensate for these disorders, patients aim their gaze toward the visual target by horizontal saccadic eye movements (Figures 1, 2, 3B, 4A,B). Because saccadic eye movements have a minimum amplitude, before a saccade commences, the gaze must be aimed away from the target by more than the saccade's minimum amplitude. The saccade amplitude is correlated with that of the distance between the very center of the fovea and the image of the visual target on the retina (DBFIR). To ensure that the DBFIR is large enough, the slow phase eye movement is implemented. The alternate appearance of saccadic eye movement and its slow phase constitutes IN. The idea that the slow phase eye movement is implemented to ensure that the DBFIR is large enough, is based on two grounds. First, the shape of the horizontal slow phase of nystagmus is an exponential function. In fact, when a small DBFIR is caused, a horizontal saccadic eye movement needs to be induced to compensate for the small DBFIR. However, when the DBFIR is less than the saccade's minimum amplitude, the patients cannot compensate for it by horizontal saccadic eye movement. Therefore, patients must increase the DBFIR by using the horizontal slow phase eye movement, similar to a positive-feedback system. As a result, the horizontal slow phase of nystagmus becomes exponential (Z component in Figure 1B). Second, the timing of the directional change of the nystagmus when the direction of the visual target movement changes. When a target moves horizontally in one direction, by setting the direction of the horizontal slow phase eye movement in the opposite direction to that of the target movement, the DBFIR is large enough and made effectively. This setting is the source of reversed OKN response. That is, when the target moves in the opposite direction, while that of the nystagmus does not change, i.e., when the horizontal direction of the slow phase of nystagmus becomes the same as that of the target's movement. Two methods are required to make the DBFIR larger during the slow phase of nystagmus. One is to set the horizontal slow phase eye velocity to a very small value so that the DBFIR becomes larger, thereby ensuring the gaze line lies behind the target's movement. The other is to set the horizontal slow phase eye velocity to a very high value so that the DBFIR becomes larger, thereby ensuring the gaze line lies ahead of the target's movement. Because of its low velocity, immediately after the target's direction changes, it is difficult to set the value of the horizontal slow phase eye velocity so that it is lower than the velocity of the target's movements. Therefore, the only option is to use the latter method. In fact, this method was used by the patient in practice, as shown in the parts indicated by the white arrow head in Figures 2B, 3B, 5A. However, because the target's velocity gradually increases and closely approaches the value of the horizontal slow phase eye velocity, increasing the DBFIR by making the gaze line lies ahead of the target's movement becomes difficult. Therefore, the nystagmus direction needs to be reversed. In practice, as shown by black arrow heads in Figures 2B, 3B, 5A, the nystagmus direction was reversed 90° after the target's direction changed where the target's velocity reached a maximum. As shown in Figures 2A,B, patient 1 showed two types of waveforms; in one type the direction of nystagmus was constant (Figure 2A) and in the other type the direction of nystagmus was variable (Figure 2B). We explained the reason why different waveforms could be observed in the same individuals as follows. According to our theory, direction alternating nystagmus as shown in Figure 2B could make DBFIR effectively more than the direction constant nystagmus as shown in Figure 2A. Therefore, the patient should use the direction alternating nystagmus when the test shown in Figure 2A. Because in order to change the direction of nystagmus effectively he had to know the timing when the velocity of leftward movement of the target became maximum, patient 1 had to learn the timing. At first, he was performed 0.3 Hz horizontal smooth pursuit test and the data shown in Figure 2A was obtained. It is thought that he used direction alternating nystagmus when 0.1 Hz horizontal smooth pursuit test as shown in Figure 2B because he learned the timing during the 0.3 Hz horizontal smooth pursuit test.

For the Y component, functions of both smooth pursuit (Figure 4C) and OKN (Figures 4D,E) were relatively maintained. However, in Z component, nystagmus was always observed even though the target did not move horizontally (Figures 4C–E). This indicates that when participants gazed at a vertically moving target by horizontal saccadic eye movement. Based on this result, patients can be considered to follow the vertical component of a target's movement by smooth pursuit (or slow phase eye movement) and the horizontal component by horizontal saccadic eye movement. Therefore, in Z component, nystagmus was evident when gazing at a still or moving target, and the patient could catch the target movement from just beside by the horizontal saccadic eye movement. In practice, during the diagonal smooth pursuit test (Figure 5A) and the diagonal OKN test (Figures 5B–D), normal smooth pursuit and slow phase eye movement were observed in Y component and nystagmus was observed at the end of the fast phase following the horizontal component of the target movement in Z component. This idea is demonstrated in the attached video (Movies 1, 2).

The idea above can also explain why nystagmus in patients with IN intensifies when tracking a visual target carefully and why it weakens in darkness (19, 20) or when eyes are closed (4). Because they can track a visual target by horizontal saccadic eye movement, the more carefully they watch a target, the more the target can be seen at the very center of the fovea, which needs induction of horizontal saccadic eye movement. Therefore, when tracking a target carefully, the frequency of horizontal saccadic eye movement must be increased, i.e., the frequency of nystagmus needs to be increased. For this to happen, the slow phase eye velocity must also be increased, resulting in nystagmus intensification. Because patients track a visual target by horizontal saccadic eye movement, this movement is not induced without a target, such as when in darkness and closed eyes conditions. As a result of not inducing horizontal saccadic eye movement, the frequency of nystagmus decreases and weakens in darkness and when eyes are closed. In practice, in dark conditions, no clear nystagmus was observed (Figure 6A). If our idea is accurate, nystagmus should have been absent in both darkness and light when no visual target existed. In practice, even in light, when the patient was looking at a white screen no visual target, no clear nystagmus was observed (Figure 6B). As shown by Richards and Wong (19), previous theories regarding the jerky type of IN depended on the presupposition that the increasing velocity slow phase was unnecessary eye movement and an obstacle for the clear vision, the “corrective saccade” compensating the unnecessary eye movement. Our new theory holds that the increasing slow phase is a necessary eye movement for making enough DBFIR to induce saccade. The finding that no clear nystagmus was observed in absence of visual target or in darkness, supports more our theory than previous ones.

Therefore, saccadic eye movement is essential to visualize a target in patients with IN. We also examined whether the characteristic of their horizontal saccadic eye movement was normal despite having a disordered horizontal smooth pursuit eye movement. In healthy participants, a larger amplitude of saccadic eye movement requires the saccadic eye movement to be of higher velocity (21). In this study, we investigated the relationship between the amplitude of horizontal saccadic eye movement and the maximum velocity of horizontal saccadic eye movement from five healthy participants and five patients. The relationship observed in patients (Figure 6F) was the same as that seen in healthy participants (Figure 6E). The relationship was normal. Harrison et al. also showed that quick phases of IN are programmed similarly to healthy subjects' saccadic eye movements (22).

In the healthy participants, during rotation, the VOR worked as an ocular gyroscope generating eye rotations to compensate for head rotation so that images of the outside world could be seen as still on the retina (23). Because VOR gain (eye velocity divided by head velocity) in darkness is lower than the ideal value of 1, VOR alone is inadequate to stabilize gaze during head rotation. The optokinetic system supplies insufficient eye movement induced by VOR (24). As shown above, the horizontal slow phase of the patients' optokinetic system with IN did not work. Therefore, patients with IN may use one of two ways to follow images of the outside world during rotation. One way is to set the VOR gain to 1. Using this method, patients can follow the image of the outside world during rotation by VOR alone. In practice, as shown in Figure 7B and the parts shown by black stars in Figure 7D, during the first half of the slow phase of nystagmus, the slow phase eye velocity generally followed on the gray line that represented the relative outside world movement. That is, the VOR gain was 1 even though the eye velocity of the last half of the slow phase of each nystagmus increased or decreased dramatically. The other way is to set the VOR gain to 0. This idea may seem unconventional. As described above, patients with IN were able to track a visual target using horizontal saccadic eye movement and to induce the saccadic eye movement needed large DBFIR. If the VOR gain gets close to 1, the eye can follow the image of the outside world by halves. As a result, they cannot follow the relative movement of the outside world perfectly, nor can they make the DBFIR large enough. If the VOR gain is 0, the DBFIR can be increased to as high an amplitude as they rotate because nonfunctional VOR fixes the eye position of the head. In practice, as shown in the parts other than those shown by black stars in Figure 7D, the slow phase eye velocity was very low, i.e., the low VOR gain value. Therefore, for patients with IN, the best VOR gain is 1, followed by a VOR gain of 0. The worst VOR gain approaches 1, e.g., 0.9, or 0.8. As shown in Figure 8, even during rotation in light; i.e., during the visuo-vestibular interaction test, patients used VOR gain values of 1 and those of low value. At the first part of the slow phase of each nystagmus during the whole rotation of patient 2 and during rightward rotation of patient 1, eye movement followed the relative movement of the outside world well, i.e., the VOR gain was 1. During leftward rotation of patient 1, the slow phase eye velocity was low (shown by black arrow heads), i.e., the low VOR gain value, and horizontal saccadic eye movement was used to follow the outside world movement.

Halmagyi et al reported the mechanisms of reversed OKN (5). They explained that the cause of reversed OKN was the shift in the predominant direction of gaze in some cases and the shift of the null zone induced by OKN stimulation in other cases. Therefore, the mechanisms differed depending on cases. As the mechanism of reversed OKN, we proposed only one mechanism that the slow phase of which direction is opposite to the stimulation plays the important role of making DBFIR effectively to induce saccade. And, by using the quite same mechanism, we can also explain why attempts to fixate upon an object accentuate nystagmus that is suppressed in darkness as shown above. Halmagyi et al also proposed that the reversed OKN was caused by abnormal decussation of temporal retinal fibers (5). More, Huang et al proposed the reversed OKN was caused by the ipsilateral projection of the retinal ganglion cell in zebrafish mutant belladonna of that OKR phenotype (25). Accordingly, a model exists of reversed OKN response where the detected retinal slip velocity is processed inversely. As shown in Figures 2, 3B, 5A, the velocity of eye movement was not always reversed. We think that in patients with a horizontal slow phase eye movement dysfunction, a reversed OKN response is not caused by an incorrect process of detecting retinal slip velocity, but by the strategy of following the OKN stimulation by horizontal saccadic eye movement.

Our theory is supported by evidence that IN is rarely present at birth, but often develops during infancy (19, 26). We think that IN appears in patients with dysfunctional gaze fixation and smooth pursuit after they acquire the ability to follow a target's movement by saccadic eye movement.

In conclusion, IN is essential for patients with disordered gaze folding, smooth pursuit and OKN, and without obstacle for getting clear vision. In patients with IN, during rotation, in the first half of the slow phase of nystagmus, their VOR works perfectly and they are able to follow the relative outside world movement. However, during the last half, their VOR is almost non-functional and the slow phase works to create enough DBFIR as preparation to induce horizontal saccadic eye movement for catching up with the outside world.

## Author contributions

TI substantial contributions to conception, design, acquisition of data, analysis and interpretation of data, and drafting of the article. YT and TO substantial contributions to acquisition of data. KH-S substantial contributions to interpretation of data. NT substantial contributions to interpretation of data and drafting of the article. KK, TF, MH, YM, and KN substantial contributions to acquisition of data. BK substantial contributions to interpretation of data and drafting of the article. HI substantial contributions to study supervision.

### Conflict of interest statement

The authors declare that the research was conducted in the absence of any commercial or financial relationships that could be construed as a potential conflict of interest.

## Supplementary material

Movie 1 Patient 1 completed the smooth pursuit test, for which the visual target moved horizontally. The movie is shown at 1/8th the actual speed of movement. In the movie, the green • indicates the target's movement, and × indicates the pupil's movement. A red × indicates eye movement occurring during the fast phase of nystagmus, and a yellow × indicates eye movement occurring during the slow phase of nystagmus. This movie shows that the patient followed the target movement using saccadic eye movement. The analyzed data are shown in Figure 2A.

Movie 2 Patient 1 completed the smooth pursuit test, for which the visual target moved vertically. The movie is shown at 1/8th the actual speed of movement. In this movie, the green •, red ×, and yellow × bear the same meanings as those in Movie 1. This movie shows that the patient could catch up with the target movement from just beside by their horizontal saccadic eye movement. The analyzed data are shown in Figure 4C.
